# Transverse cardiac slicing and optical imaging for analysis of transmural gradients in membrane potential and Ca^2+^ transients in murine heart

**DOI:** 10.1113/JP276239

**Published:** 2018-07-26

**Authors:** Q. Wen, K. Gandhi, Rebecca A. Capel, G. Hao, C. O'Shea, G. Neagu, S. Pearcey, D. Pavlovic, Derek A. Terrar, J. Wu, G. Faggian, Patrizia Camelliti, M. Lei

**Affiliations:** ^1^ Institution of Cardiology Union Hospital Tongji Medical College Huazhong University of Science and Technology Wuhan China; ^2^ Medical School University of Verona Verona Italy; ^3^ Department of Pharmacology University of Oxford Oxford UK; ^4^ Key Laboratory of Medical Electrophysiology of Ministry of Education Collaborative Innovation Center for Prevention and Treatment of Cardiovascular Disease/Institute of Cardiovascular Research Southwest Medical University Luzhou 6400 China; ^5^ Institute of Cardiovascular Sciences University of Birmingham Birmingham UK; ^6^ School of Biosciences and Medicine University of Surrey Guildford UK

**Keywords:** cardiac slices, optical imaging, murine heart, electrophysiological heterogeneity

## Abstract

**Key points:**

- A robust cardiac slicing approach was developed for optical mapping of transmural gradients in transmembrane potential (*V*_m_) and intracellular Ca^2+^ transient (CaT) of murine heart.
- Significant transmural gradients in *V*_m_ and CaT were observed in the left ventricle.
- Frequency‐dependent action potentials and CaT alternans were observed in all ventricular regions with rapid pacing, with significantly greater incidence in the endocardium than epicardium.
- The observations demonstrate the feasibility of our new approach to cardiac slicing for systematic analysis of intrinsic transmural and regional gradients in *V*_m_ and CaT.

**Abstract:**

Transmural and regional gradients in membrane potential and Ca^2+^ transient in the murine heart are largely unexplored. Here, we developed and validated a robust approach which combines transverse ultra‐thin cardiac slices and high resolution optical mapping to enable systematic analysis of transmural and regional gradients in transmembrane potential (*V*_m_) and intracellular Ca^2+^ transient (CaT) across the entire murine ventricles. The voltage dye RH237 or Ca^2+^ dye Rhod‐2 AM were loaded through the coronary circulation using a Langendorff perfusion system. Short‐axis slices (300 μm thick) were prepared from the entire ventricles (from the apex to the base) by using a high‐precision vibratome. Action potentials (APs) and CaTs were recorded with optical mapping during steady‐state baseline and rapid pacing. Significant transmural gradients in *V*_m_ and CaT were observed in the left ventricle, with longer AP duration (APD_50_ and APD_75_) and CaT duration (CaTD_50_ and CaTD_75_) in the endocardium compared with that in the epicardium. No significant regional gradients were observed along the apico‐basal axis of the left ventricle. Interventricular gradients were detected with significantly shorter APD_50_, APD_75_ and CaTD_50_ in the right ventricle compared with left ventricle and ventricular septum. During rapid pacing, AP and CaT alternans were observed in most ventricular regions, with significantly greater incidence in the endocardium in comparison with epicardium. In conclusion, these observations demonstrate the feasibility of our new approach to cardiac slicing for systematic analysis of intrinsic transmural and regional gradients in *V*_m_ and CaT in murine ventricular tissue.

## Key points

- A robust cardiac slicing approach was developed for optical mapping of transmural gradients in transmembrane potential (*V*_m_) and intracellular Ca^2+^ transient (CaT) of murine heart.
- Significant transmural gradients in *V*_m_ and CaT were observed in the left ventricle.
- Frequency‐dependent action potentials and CaT alternans were observed in all ventricular regions with rapid pacing, with significantly greater incidence in the endocardium than epicardium.
- The observations demonstrate the feasibility of our new approach to cardiac slicing for systematic analysis of intrinsic transmural and regional gradients in *V*_m_ and CaT.

## Introduction

Because of its feasibility for genetic modification, the mouse has been the most popular animal species for modelling human disease conditions and for mechanistic research, as well as therapeutic exploration, despite distinct differences in its cardiac electrophysiological properties compared to the human heart, including marked differences in heart size, heart rates and action potential waveforms. The mouse is also the second mammalian species, after humans (Lander *et al*. 2001), in which a substantial amount of genomic information has been analysed (Waterston *et al*. 2002). Within the cardiovascular research community, the mouse has been widely used for exploring molecular, cellular and systemic mechanisms underlying inherited and acquired ventricular arrhythmic diseases (Nerbonne, 2014). As transgenic technology has advanced, mutagenesis has become much easier to carry out in mice and an increasing number of genetically modified mouse systems have been generated for the study of cardiac arrhythmias (Sabir *et al*. 2008). The models include ion channelopathies with minimal structural abnormalities, and those of structural heart disease (Choy *et al*. 2016). The former group includes catecholaminergic polymorphic ventricular tachycardia (CPVT) (Wehrens *et al*. 2003), the long (Wu *et al*. 2012) and short QT syndromes (LQTS and SQTS), and Brugada syndrome (BrS) (Papadatos *et al*. 2002; Choy *et al*. 2016). The latter group includes several types of cardiomyopathies, such as arrhythmogenic right ventricular dysplasia (ARVD) (Asano *et al*. 2004), dilated cardiomyopathy (DCM) and hypertrophic cardiomyopathy (HCM) (Choy *et al*. 2016).

Surprisingly, despite the popularity of mouse models in heart research, little is known about murine heart transmural and regional electrophysiological heterogeneities such as action potential and Ca^2+^transient characteristics. These are vitally important for phenotypic and mechanistic research into cardiac disease conditions, in particular cardiac arrhythmias. In this species, the transmural and regional electrophysiological heterogeneities have been largely unexplored.

Thin tissue slices, a well‐established experimental preparation for electrophysiological studies of the brain, have recently emerged as a promising model for cardiac electrophysiology investigations. Thin slices, prepared from the ventricle of a number of different species (Halbach *et al*. 2006; Bussek *et al*. 2009; Camelliti *et al*. 2011; Wang *et al*. 2014), have been shown to retain structural and functional properties of the native myocardium, including tissue architecture, cell type ratio, cell distribution, cell–cell electrical and mechanical coupling, and extracellular matrix (Bussek *et al*. 2009; Camelliti *et al*. 2011). Importantly, cardiac slices exhibit similar electrophysiological characteristics to the intact heart and respond to the application of pharmacological compounds similarly to the whole heart (Camelliti *et al*. 2011; Himmel *et al*. 2012), thus providing a promising experimental model for electrophysiology, Ca^2+^ handling, and drug action investigations.

Most previous electrophysiology studies on cardiac slices have employed low spatial resolution recording methods, including patch clamp (Burnashev *et al*. 1990), sharp electrodes (Halbach *et al*. 2006; Bussek *et al*. 2009; Himmel *et al*. 2012) and low‐density multi‐electrode arrays (Bussek *et al*. 2009; Camelliti *et al*. 2011). Higher spatial resolution methods, such as optical mapping, provide exciting opportunities to monitor dynamic cellular electrophysiological events as they occur in real time (Wang *et al*. 2015; Kang *et al*. 2016). Furthermore, prior studies have been limited to the analysis of sub‐epicardial (Wang *et al*. 2015) or sub‐endocardial tangentially cut slices (Kang *et al*. 2016), or a few transmurally cut slices (Bussek *et al*. 2009), an approach inadequate for accurate capture of regional and transmural cardiac electrophysiological properties. A method for systematic analysis of regional and transmural electrophysiological heterogeneities across the whole ventricles in mouse is currently missing.

In order to address the issues described above, in the present study we developed a feasible transverse slicing method and combined it with a high‐throughput optical imaging technique as a new approach for studying cellular electrophysiology of murine heart in intact sliced ventricular tissue. These transverse slices were cut at right angles to the long axis of the heart. Our approach enables, for the first time, the use of a series of slices prepared from ventricle to measure transmembrane potential (*V*_m_) and intracellular Ca^2+^ transient (CaT) with high temporal and spatial resolution, allowing (i) comparison of successive slices which form a stack representing the original geometry of the heart; (ii) profiling of the transmural and regional gradients in *V*_m_ and CaT in the ventricle; (iii) characterisation of transmural and regional profiles of action potential and CaT alternans.

## Methods

### Ethical approval

All animal experiments were performed on adult mice (CD1, 12–20 weeks old) in accordance with the United Kingdom Animals (Scientific Procedures) Act 1986 and were approved by the University of Oxford Pharmacology ethical committee (approval ref. PPL: 30–3340) and the national guidelines under which the institution operates. All mice used in this study were maintained in a pathogen‐free facility at the University of Oxford. Mice were given *ad libitum* access to food and water. The authors confirm that they have taken all steps to minimise the animals’ pain and suffering. Our work complies with the journal policy and regulations (Grundy, 2015).

### Heart isolation

Mice were killed by cervical dislocation in accordance with Schedule 1 killing method. Hearts were rapidly excised and Langendorff‐perfused with oxygenated Krebs solution (containing in mM: NaCl 119, NaHCO_3_ 25, sodium pyruvate 1.8, KH_2_PO_4_ 1.2, KCl 4.7, MgCl_2_ 1.0, CaCl_2_ 1.8, and glucose 10; equilibrated with 95% O_2_/5% CO_2_, pH 7.4) at 37°C and at constant rate of 3.5–4 ml/min for 10 min. To prevent formation of blood clots in the coronary circulatory system, animals were treated with heparin (200 units) under non‐recovery terminal general anaesthesia by injection of an overdose of 1.2% Avertin solution (0.5∼0.8 ml i.p. (2,2,2‐tribromoethanol, Sigma‐Aldrich Poole, Dorset, UK) prior to killing and hearts were injected with streptokinase (400 units) via the coronary system after organ excision according to approved procedure by the Home Office.

### Dye loading

Fluorescent dyes were loaded via the coronary circulation during Langendorff perfusion, applied by injection into the aortic cannula. The voltage sensitive dye RH237 (Thermo Fisher Scientific, UK) was delivered as a 15 μl bolus of 2 mM concentration, injected into the cannula in small volume steps over a period of 5 min. The Ca^2+^ dye Rhod‐2 AM (Thermo Fisher Scientific, UK) was administered as a 50 μl bolus (stock solution: 1 mg/ml in DMSO) over a 5 min period, and recirculated for 45 min in the presence of 0.5 mM probenecid. After dye loading, hearts were perfused with Krebs solution containing 10 μM blebbistatin, a myosin II inhibitor used to stop contractions and avoid movement artefacts during optical mapping recordings.

### Slice preparation

Table 1 summarises the slice preparation procedure. Hearts were removed from the Langendorff set‐up and dissected in cold (4°C) oxygenated (99.5% O_2_) Tyrode solution (in mM: NaCl 140; KCl 4.7; glucose 10; HEPES 10; MgCl_2_ 1; CaCl_2_ 1.8; pH 7.4) containing the excitation–contraction uncoupler 2,3‐butanedione monoxime (BDM, 10 mM). Atria and valves were removed using a sharp scalpel blade, leaving the ventricles intact. Ventricles were glued base‐down (histoacryl tissue adhesive; Braun, Melsungen, Germany) onto a pre‐made 4% agar block, previously fixed onto the vibratome specimen holder (VF‐300 vibrating microtome; Precisionary Instruments Inc., Greenville, USA). Ventricles were subsequently embedded in 4% low‐melt agarose and cooled on ice at 4°C. The specimen holder with agarose‐embedded ventricles was mounted onto the stage of the vibratome filled with cold (4°C) oxygenated (99.5% O_2_) Tyrode solution containing BDM. Short‐axis slices were cut at a thickness of 100 μm, 300 μm or 500 μm, using a razor blade at a speed of 0.02 mm/s and vibration frequency of 82 Hz. After initial experiments aiming to determine the best slice thickness for dye loading, dye retention and optical mapping recordings, short‐axis slices of 300 μm thickness were prepared from the entire ventricles (from the apex to the base) for the study of regional and transmural *V*_m_ and Ca^2+^ properties. As summarised in Table 1, the total cold ischaemic time during slicing is between 1.5 and 4 min. Slices were then transferred to recovery solutions for recovery in a pre‐incubation chamber filled with Krebs solution containing 10 μM blebbistatin at room temperature for 30 min before electrophysiological investigations. To prevent tissue from curling, slices were collected on Sylgard blocks and held in position using a nylon mesh in the pre‐incubation chamber.

**Table 1:** Summary of the slicing and imaging protocol

	Dye loading	Perfusion	Slicing	Recovery 1	Recovery 2	Recovery 3	Pre‐imaging	Imaging
Temperature	37°C	37°C	4°C	4°C	RT	RT	37°C	37°C
Solution	Krebs	Krebs	Tyrode	Tyrode	Krebs	Krebs	Krebs	Krebs
BDM (10 mM)	No	No	Yes	Yes	No	No	No	No
Blebbistatin (10 μM)	No	Yes	No	No	Yes	Yes	Yes	Yes
Ca^2+^	1.8 mM	1.8 mM	1.8 mM	1.8 mM	1.8 mM	1.8 mM	1.8mM	1.8mM
O_2_	95%	95%	100%	100%	95%	95%	95%	95%
CO_2_	5%	5%	0%	0%	5%	5%	5%	5%
Duration	5–7 min	20 min	1.5–4 min	10 min	10 min	5 min	3 min	<1 min

RT, room temperature; Krebs, Krebs‐Henseleit solution; Tyrode, Tyrode solution.

### Optical mapping

**Figure 1:**
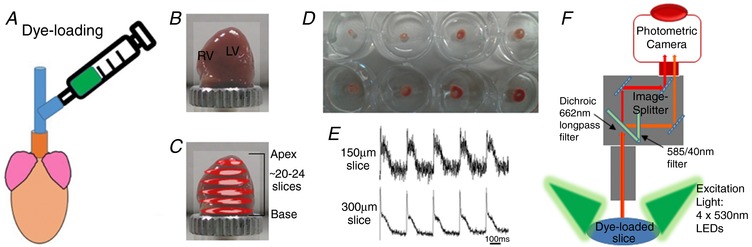
Optimisation of murine cardiac slice methodology *A*, voltage (RH237) or calcium (Rhod‐2 AM) dyes were loaded via coronary circulation using the Langendorff perfusion system. *B*, ventricles were embedded in 4% low‐melt agarose and mounted onto the vibratome specimen holder. *C*, up to 24 transverse slices were cut from the apex to the base in ice‐cold BDM Tyrode solution. *D*, slices were recovered at room temperature in Krebs solution containing 10 μM blebbistatin for 30 min, before optical mapping studies. *E*, *V*_m_ signals in slices cut at 150 μm and 300 μm thickness. *F*, optical mapping setup with four 530 nm excitation LEDs and a 128 × 128 EMCCD camera.

Slices were electrophysiologically assessed with the optical mapping method, using a custom‐designed optical mapping system equipped with an EMCCD camera (Evolve 128 Photometrics, Tucson, AZ, USA). Figure 1 *F* shows a schematic diagram of the mapping set‐up. Slices were kept in Krebs solution containing 10 μM blebbistatin at 37°C during imaging. Four 530 nm LEDs were used for excitation of the Ca^2+^‐sensitive dye Rhod‐2. CaT fluorescence was collected at 585 ± 40 nm. For *V*_m_ signals, four 530 nm LEDs were used for excitation of RH237, and emission was collected using a 662 nm long pass filter. *V*_m_ and CaT measurements were taken at maximal resolution (128 × 128 pixels; pixel area 47 × 47 μm) at a rate of 1000 frames/s. Slices were electrically stimulated with bipolar pulses of 2 ms duration, at voltages 1.5 times above threshold (5–10 V) and initial frequency of 2 Hz. For AP and CaT alternans investigations, slices were stimulated at frequencies of 2, 4, 8 and 16 Hz.

### Data analysis

AP duration (APD) and CaT duration (CaTD) were analysed using OPTIQ (program developed by Dr Burton, University of Glasgow). AP and CaT signals were filtered using a Gaussian spatial filter (radius 2 pixels) before relevant parameters were extracted. APD was measured as the time from the upstroke to 50% and 75% repolarisation (APD50 and APD75). Similarly, the CaTD was measured as the time from the upstroke to 50% and 75% recovery (CaTD50 and CaTD75). AP and CaT alternans are defined as the electrical or calcium transient discordant in duration or in amplitude. These phenomena depend on the underlying instability mechanisms (*V*_m_‐driven or CaT‐driven) and the coupling between AP and CaT alternans cycling.

For assessment of conduction velocity (CV), the multi‐vector polynomial method (Bayly *et al*. 1998) was used within bespoke analysis software, ElectroMap (O'Shea *et al*. 2017). Following Gaussian filtering (3 × 3 pixel area, σ = 1.5), activation maps, generated from measuring the time of the depolarisation midpoint, were spatially segmented into regions of 5 × 5 pixels. A polynomial surface was then fitted to local activation times to describe propagation in the area, and local CV quantified as the gradient vector of the polynomial surface. Mean CV was then calculated from the local CVs across the tissue slice.

### Statistical analysis

Transmural and regional APD/CaTD distributions were analysed using a Student's paired *t* test or one‐way ANOVA with *post hoc* Tukey's test and repeated measures correlation among tissue regions. Regional frequency‐dependent AP/CaT alternans and arrhythmia heterogeneity were analysed using a chi‐squared test. A value of *P *< 0.05 was considered statistically significant. Values are expressed as means ± SEM.

## Results

### Optimisation of murine transverse ventricular slices for *V*_m_ and CaT measurements

**Figure 2:**
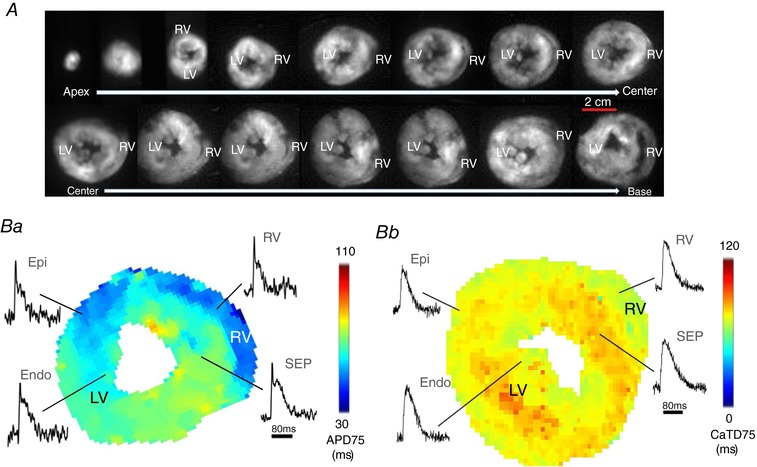
*V*_m_ and CaT measurements in murine ventricular slices *A*, bright field images of transverse ventricular slices (300 μm thick) prepared from both ventricles of a mouse heart (from the apex to the base). Scale bar: 2 cm. *B*, AP duration (APD; *Ba*) and CaT duration (CaTD; *Bb*) maps generated from a slice prepared from the central region of the ventricles, with raw AP and CaT signals acquired from the epicardium (Epi) and endocardium (Endo) of the left ventricle, the ventricular septum (SEP) and the right ventricle (RV). APD scale bar: 80 ms.

Adult murine ventricular slices have been previously developed for electrophysiology applications (Halbach *et al*. 2006), but optimisation of the slice preparation to enable systematic analysis of *V*_m_ and CaT across the whole murine ventricles has yet to be achieved. Figure 1 illustrates the methodology implemented in this study. Critical steps included determination of an efficient dye loading method, a slice thickness able to maintain intact tissue morphology and generate optimal voltage and calcium signals, and a recovery period necessary to reach stable signals after slice preparation. As shown in Fig. 1, to achieve optimal dye loading, the voltage dye RH237 or Ca^2+^ dye Rhod‐2 AM were loaded through the coronary circulation using the Langendorff perfusion system. Prevention of blood clot formation was essential for optimal dye loading and was achieved by initial injection of heparin (200 units) before killing, and subsequent injection of streptokinase (400 units) directly into the coronary system after heart excision. Ventricles were embedded in low‐melting temperature agarose to provide structural support to the tissue during sectioning. Slices were cut at right angles to the long axis of the heart from the apex to the base, and thicknesses from 100 μm to 500 μm were tested, with 300 μm found to be the best thickness to achieve optimal recordings for both *V*_m_ and CaT. Slices less than 300 μm thick showed markedly lower *V*_m_ and CaT signals (Fig. 1 *E*), while slices thicker than 300 μm suffered from poor oxygenation. After preparation, slices were pre‐incubated at room temperature in Krebs solution containing 10 μM blebbistatin for an optimal recovery time of 30 min, prior to electrophysiological assessment using a custom‐designed optical mapping system (Fig. 1 *F*). Figure 2 shows viable slices obtained with the optimised protocol, summarised in Table 1, and representative *V*_m_ and CaT signals from different regions, illustrating the robustness of this preparation for murine research. We monitored the ‘rundown’ of the *V*_m_ and CaT signals for up to 4 h, the average signal ‘rundown’ is less than 25%. Our experiments usually finished within 3 h.

### Transmural and regional characteristics of *V*_m_

After successful optimisation of the methodology, we tested the feasibility of combining murine slices with high resolution optical mapping for systematic transmural and regional profiling of *V*_m_ and CaT across the murine ventricles. Transverse slices (300 μm thick) were prepared from the entire ventricles (from apex to base; Fig. 2 *A*), and AP duration (APD) and CaT duration (CaTD) were mapped with high spatial and temporal resolution. Examples are shown in Fig. 2 *B* corresponding to slice 10 shown in Fig. 2 *A*.

**Figure 3:**
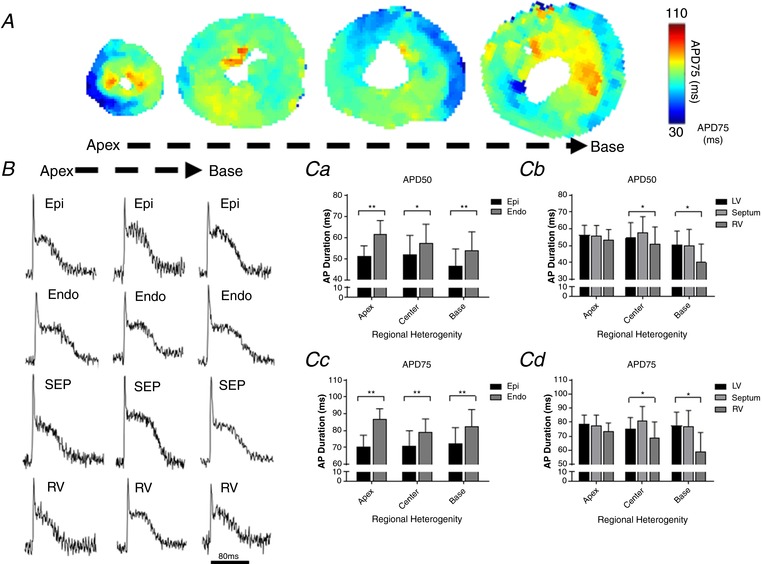
Transmural and regional distribution of AP duration across the murine ventricles *A*, representative maps of AP duration (APD75) at 2 Hz pacing frequency (500 ms pacing cycle length) recorded from apex to base in transverse ventricular slices. *B*, typical optical AP recordings (unfiltered signals) obtained from different regions of the murine ventricles at 2 Hz pacing frequency. Scale bar: 80 ms. *C*, quantitative summary of transmural and regional APD50 (*Ca* and *Cb*) and APD75 distribution (*Cc* and *Cd*) at 2 Hz pacing (*n = *5 hearts; ^**^ *P < *0.01; ^*^ *P < *0.05). Values expressed as means ± SEM. Epi, epicardium; Endo, endocardium; SEP, septum; RV, right ventricle; LV, left ventricle.

Figure 3 summarises the distribution of APD at 50% repolarisation (APD50) and 75% repolarisation (APD75) across the entire murine ventricles at 2 Hz pacing frequency. Figure 3 *A* and *B* show typical APD75 colour‐coded maps and action potentials from a series of transverse ventricular slices, suggesting the presence of APD heterogeneities in the mouse ventricles. An important advantage of the cardiac slice model is the ability to study any region of the mouse ventricle and thus simultaneously investigate transmural, apico‐basal (apex–base), and interventricular APD gradients. As shown in Fig. 3 *C*, there are significant transmural gradients in APD50 and APD75 in the left ventricle at 2 Hz pacing frequency (Fig. 3 *Ca* and *c*), with the shortest APDs occurring in the epicardium and the longest APDs occurring in the endocardium (APD50: Epi 50 ± 7 ms, Endo 58 ± 8 ms, *P < *0.01; APD75: Epi 71 ± 8 ms, Endo 83± 8 ms, *P* = 0.0001, paired *t* test; *n = *5 hearts). Differences between longest and shortest APD (transmural APD dispersion) in LV free wall were 8 ± 1 ms at APD50 and 11± 1 ms at APD75. Importantly transmural heterogeneities were not restricted to a particular region of the left ventricle, but were present along the entire left ventricle free wall from the apex to the base (Fig. 3 *Ca* and *c*), with the highest transmural APD dispersion occurring in the apical region (apex transmural dispersion: 16 ± 1 ms at APD75; *P < *0.05, one‐way ANOVA comparing apex‐centre‐base transmural dispersion).

In addition to transmural APD distribution, cardiac slices allowed us to investigate APD gradients across the apico‐basal axis of the left ventricle free wall. As shown in Fig. 3 *Ca* and *c*, there is no significant apico‐basal gradient in APD50 and APD75 in either epicardium or endocardium (*P *> 0.05, one‐way ANOVA with *post hoc* Tukey's test; *n = *5 hearts).

Finally, as each slice contains left ventricle free wall, right ventricle and septum, we were able to assess interventricular APD gradients. As shown in Fig. 3 *Cb* and *d*, there are significant interventricular differences in APD50 and APD75 at the base and centre, but not at the apex. APD was shorter in the right ventricle in comparison to the left ventricle and the septum, in the basal and centre regions (*P < *0.05, one‐way ANOVA with *post hoc* Tukey's test; *n = *5 hearts), with interventricular APD75 dispersion of 23 ± 6 ms and 13 ± 3 ms, respectively.

### Transmural and regional characteristics of CaT

**Figure 4:**
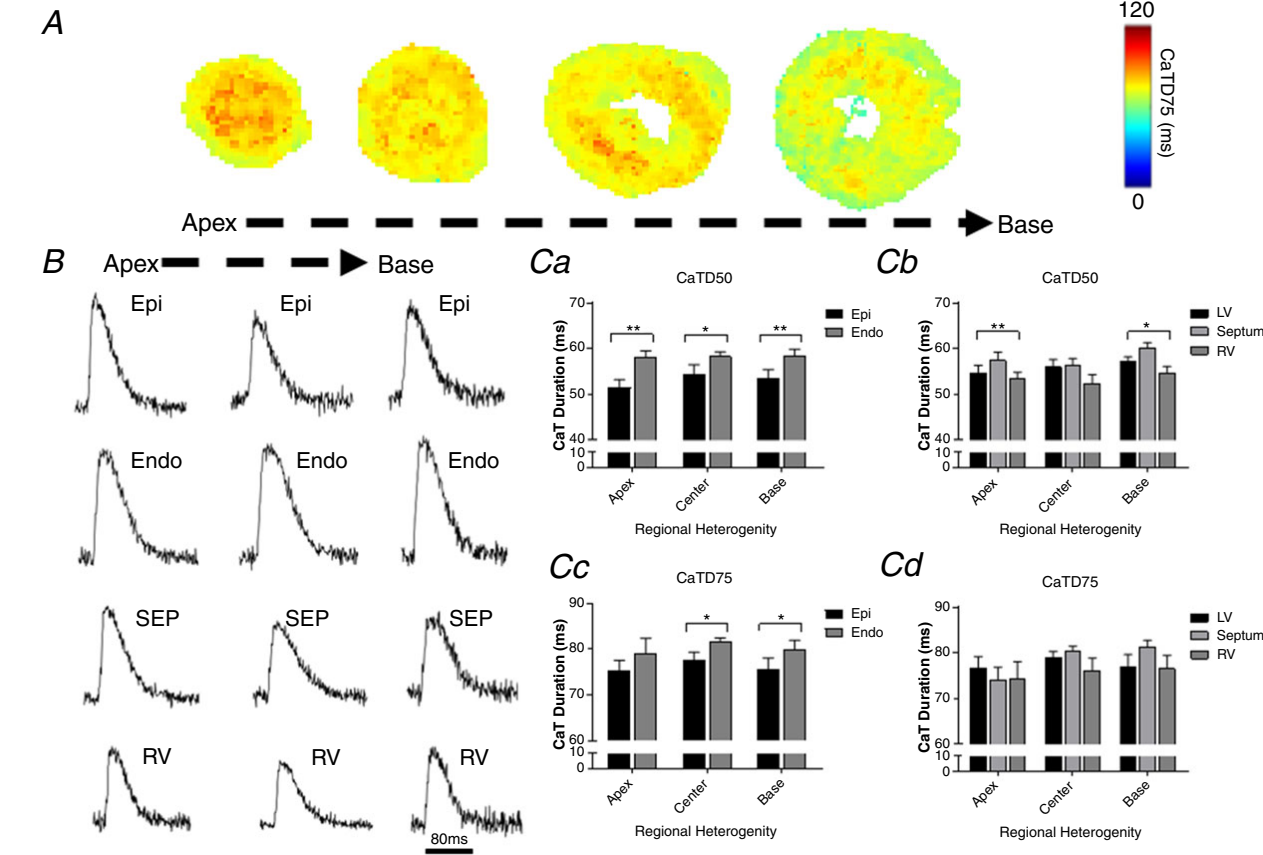
Transmural and regional distribution of CaT duration in the murine ventricles *A*, representative maps of CaT duration (CaTD75) at 2 Hz pacing frequency (500 ms pacing cycle length) recorded from apex to base in transverse ventricular slices. *B*, representative optical CaT raw traces obtained from different regions of the murine ventricles at 2 Hz pacing frequency. Scale bar: 80 ms. *C*, quantitative summary of transmural and regional CaTD50 (*Ca* and *Cb*) and CaTD75 distribution (*Cc* and *Cd*) at 2 Hz pacing (*n = *5–8 hearts; ^**^ *P < *0.01; ^*^ *P < *0.05). Values expressed as means ± SEM. Epi, epicardium; Endo, endocardium; SEP, septum; RV, right ventricle; LV, left ventricle.

Analysis of transmural and regional heterogeneity in calcium handling are summarised in Fig. 4. Figure 4 *A* and *B* show representative maps of CaT duration (CaTD) at 75% recovery (CaTD75) from apical, centre and basal ventricular mouse slices during electrical pacing at 2 Hz frequency (Fig. 4 *A*), and typical optical CaT recordings from corresponding regions (Fig. 4 *B*). As shown in Fig. 4 *C*, there are significant transmural gradients in CaTD at 50% recovery (CaTD50) in apical, centre and basal regions of the left ventricle free wall at 2 Hz pacing frequency (Fig. 4 *Ca*), with significantly longer CaTD50 in the endocardium compared to the epicardium (apex: 13% longer, *P < *0.01; centre: 8%, *P < *0.05; base: 10%, *P < *0.01; *n = *6–8 hearts). A significant transmural gradient in CaTD75 (Fig. 4 *Cc*) was also observed in the basal and centre regions of the left ventricle (*P < *0.05, paired *t* test; *n = *5–7 hearts), but not in the apical region.

We further assessed the distribution of CaTD along the apico‐basal axis of the left ventricle free wall. As shown in Fig. 4 *Ca* and *c*, no significant apico‐basal gradient in CaTD50 and CaTD75 was observed in the murine left ventricle (*P* > 0.05, one‐way ANOVA; *n = *5–7 hearts).

Finally, we compared CaTD measured in left ventricle free wall regions with CaTD recorded in right ventricle and ventricular septum regions for each tissue slice, to investigate interventricular CaTD gradients. As shown in Fig. 4 *Cb* and *d*, there are significant regional differences in CaTD50 but not CaTD75 at the apex and base, with the shortest CaTD50 occurring in the right ventricle, and an interventricular CaTD50 dispersion of 4 ± 1 ms and 6 ± 1 ms at the apex and base, respectively (*n = *6–7 hearts).

### Transmural and regional heterogeneity of frequency‐dependent AP and CaT alternans

**Figure 5:**
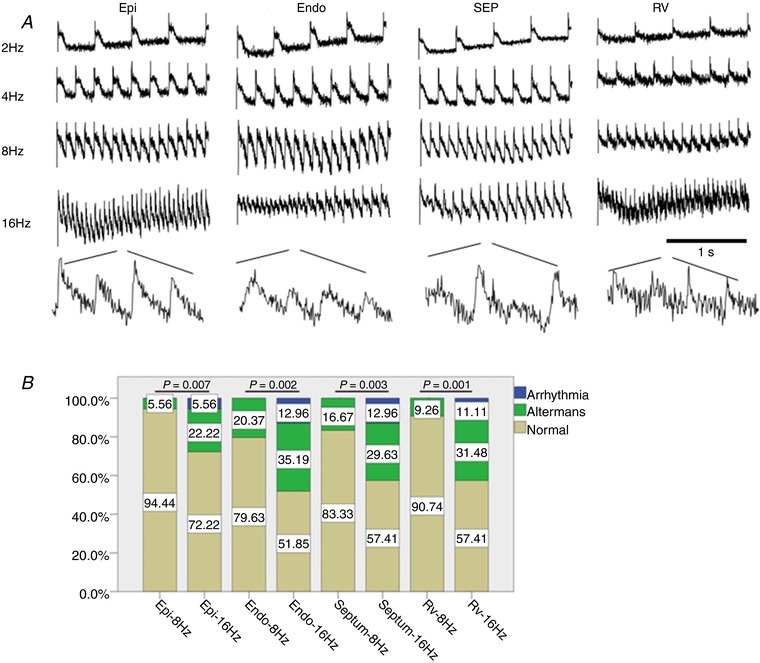
Regional and frequency‐dependent distribution of AP alternans and arrhythmic events in murine ventricular slices *A*, representative optical AP traces obtained from different regions within a ventricular slice during electrical pacing at 2, 4, 8 and 16 Hz frequency. Scale bar: 1s. *B*, transmural and regional occurrence of alternans and arrhythmias at 8 Hz and 16 Hz pacing frequency. Epi, epicardium; Endo, endocardium; SEP, septum; RV, right ventricle; LV, left ventricle. *n = *6 hearts.

**Figure 6:**
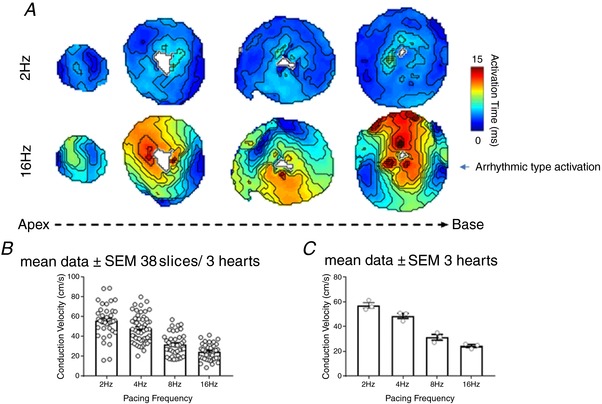
Analysis of conduction velocity (CV) *A*, activation maps of slices (apex to base) paced at 2 Hz and 16 Hz. Conduction velocity (CV) was calculated using a multi‐vector polynomial method within bespoke ElectroMap analysis software. A polynomial surface was fitted to local activation times to describe propagation in the area, and local CV quantified as the gradient vector of the polynomial surface. Mean CV was then calculated from the local CVs across the tissue slice. *B*, mean data for CV at a range of pacing frequencies calculated across all the slices (*n = *38 slices/3 hearts). *C*, mean data for CV at a range of pacing frequencies calculated across all the hearts (*n = *3 hearts).

**Figure 7:**
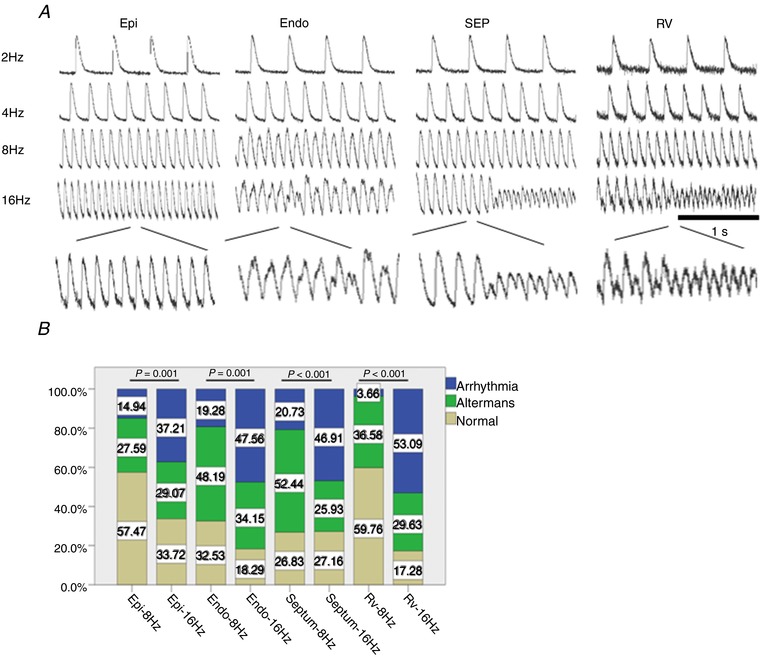
Regional and frequency‐dependent distribution of CaT alternans and arrhythmic events in murine ventricular slices *A*, representative optical CaT traces obtained from different regions within a ventricular slice during electrical pacing at 2, 4, 8 and 16 Hz frequency. Scale bar: 1s. *B*, transmural and regional occurrence of alternans and arrhythmias at 8 Hz and 16 Hz pacing frequency. Epi, epicardium; Endo, endocardium; SEP, septum; RV, right ventricle; LV, left ventricle. *n = *6 hearts.

Further experiments were carried out to assess the feasibility of using murine transverse ventricular slices and optical mapping to characterise transmural and regional differences in frequency‐dependent AP and CaT alternans and arrhythmias. Slices were paced at frequencies of 2, 4, 8 and 16 Hz, and AP and CaT were recorded at each frequency (Figs 5 *A* and 7 *A*). As shown in Figs 5 and 7, AP and CaT alternans and arrhythmic events often occurred at higher pacing frequencies of 8 Hz and 16 Hz, indicating a frequency‐dependent relationship. At a high pacing rate of 16 Hz, alternans often converted into arrhythmia. An increase in AP alternans and arrhythmic events occurred in all regions (LV, septum and RV) during incremental pacing from 8 Hz to 16 Hz frequency (Fig. 5 *B*). Transmural heterogeneities were observed in the LV free wall, with greater AP alternans and arrhythmic events in the endocardium in comparison with epicardium at both 8 Hz and 16 Hz frequencies (Fig. 5 *B*; *P < *0.05, *n = *6 hearts). There are no significant interventricular differences observed in the incidence of AP alternans, with a similar number occurring in the left ventricle, right ventricle and septum at both 8 Hz and 16 Hz frequencies. Figure 6 illustrates the activation maps generated within bespoke ElectroMap analysis software (O'Shea *et al*. 2017). A polynomial surface was then fitted to local activation times to describe propagation in the area, and local CV quantified as the gradient vector of the polynomial surface. Mean CV was then calculated from the local CVs across the tissue slice. As seen in Fig. 6, there is a clear slowing of conduction at higher pacing frequencies. This is apparent when CV data is analysed across all the slices (Fig. 6 *A*‐*C*) or across individual hearts (Fig. 6 *A*‐*C*). In some slices we analysed there is evidence of steep activation gradients and focal activity, which may contribute to the arrhythmias observed.

As shown in Fig. 7 *B*, a significant increase in CaT alternans and arrhythmic events occurred in the epicardium and endocardium of the left ventricle, in the ventricular septum and in the right ventricle with increase of pacing frequency from 8 Hz to 16 Hz. Transmural heterogeneities were also observed, with lower occurrence of CaT alternans and arrhythmic events in the epicardium in comparison with endocardium at both 8 Hz and 16 Hz frequencies (Fig. 7 *B*; *P < *0.05, *n = *6 hearts).

## Discussion

In the present study we developed and validated a transverse ventricular tissue slice model to enable systematic analysis of *V*_m_ and CaT across the whole murine ventricles. Our investigation of murine transverse slices, prepared from both ventricles (from apex to base) combined with high resolution optical mapping, revealed transmural and regional heterogeneities in both physiological APD and CaTD, and in the incidence of frequency‐dependent AP and CaT alternans in the murine ventricles.

### Optimisation of cardiac slices for *V*_m_ and CaT measurements

Cardiac slice technology has previously been employed for structural and functional studies of human biopsies and animal hearts (Halbach *et al*. 2006; Bussek *et al*. 2009; Camelliti *et al*. 2011), but application of the preparation in optical mapping studies is only starting to emerge (Wang *et al*. 2015; Kang *et al*. 2016). This report is the first to present a detailed protocol for successful preparation of transverse ventricular slices for transmural and regional profiling of *V*_m_ and CaT with high resolution optical mapping. Important steps include dye loading via Langendorff perfusion; slice cutting perpendicular to the long axis of the heart; a slice thickness of 300 μm; and a post‐cutting recovery period of 30 min.

In contrast to prior reports of cardiac slice preparation, slices were cut at right angles to the long axis of the heart (transverse slices) and not tangentially to the epicardial surface (Bussek *et al*. 2009; Camelliti *et al*. 2011). This allowed us to prepare a complete series of slices containing left ventricle, right ventricle and ventricular septum regions from apex to base. Given the curvature of the mouse ventricles and the different thickness of LV, RV and septum at the apex and base, preparing tangential slices containing well aligned cardiomyocytes covering the entire ventricular tissue, for the study of transmural and regional heterogeneities, was not feasible. Importantly our study demonstrates that transverse mouse slices are viable and show robust voltage and calcium signals. We identified slice thickness as a critical parameter for successful preparation of viable transverse slices. Previous studies have reported thicknesses from 150 to 500 μm (Pertsov *et al*. 1993; Halbach *et al*. 2006; Camelliti *et al*. 2011; Wang *et al*. 2015), but in this study we determined that 300 μm was the best slice thickness to guarantee cell viability and achieve optimal *V*_m_ and Ca^2+^ signals using optical mapping techniques. Transverse slices less than 300 μm showed significantly reduced *V*_m_ and CaT signals (Fig. 1 *E*). We suggest that, with mouse ventricular myocyte dimensions estimated as 90 μm (length) × 25 μm (width) (Toischer *et al*. 2010), overly thin slices may result in damage to the vast majority of available cells within the slice. Slices thicker than 400 μm, on the other hand, required larger electrical stimulation pulses, longer post‐cutting recovery time, and suffered from poor oxygenation and limited BDM washout. BDM, if not completely washed out, is known to have residual effects on cardiac ion channels (Coulombe *et al*. 1990). Oxygen supply, in the absence of vascular perfusion, is guaranteed by diffusion in cardiac slices. However, as the maximum diffusion distance for cardiac contracting muscle is estimated to be 150 μm (Barclay, 2005), slices thicker than 300 μm will be exposed to hypoxic conditions, resulting in limited viability.

In previous studies, post‐cutting recovery times ranging from 30 min (Burnashev *et al*. 1990) to 2 h (Yasuhara *et al*. 1996) were reported. For transverse mouse ventricular slices we determined an optimal recovery period to be 30 min. Slices cut in cold (4°C), oxygenated (99.5% O_2_) Tyrode solution containing BDM were placed in Krebs solution containing 10 μM blebbistatin at room temperature (equilibrated with 95% O_2_/5% CO_2_) to recover. The recovery time of 30 min allowed washout of BDM and returned the slices to a more physiological temperature. Our findings are consistent with previous reports indicating recovery times for adult guinea‐pig slices and neonatal rat slices of, respectively, 36 min (Wang *et al*. 2015) and 30 min (Pertsov *et al*. 1993; Davidenko *et al*. 1995).

Conduction velocity as an indicator of physiological state is shown in Fig. 6. We demonstrate that conduction velocity in our cardiac slices is comparable to conduction velocity measured in whole mouse hearts by epicardial optical mapping (Baker *et al*. 2000; Baudenbacher *et al*. 2008; Myles *et al*. 2012; Bao *et al*. 2016). As expected, higher pacing frequencies lead to reduced conduction velocities, further demonstrating physiological responsiveness of the slices (Weber *et al*. 2011).

### Cardiac slices for the study of transmural and regional heterogeneity of *V*_m_ and CaT

A critical advantage of the cardiac slice model is the ability to provide access to any region of the ventricles, thus enabling exploration of regional and transmural differences. This is particularly important for the mouse heart, given that other *in vitro* tissue preparations, such as ventricular wedges, are not feasible for small hearts.

Action potential and calcium handling heterogeneities, and their role in the generation of arrhythmias, are well documented in hearts from larger mammals (Yan *et al*. 1998; Burton & Cobbe, 2001; Laurita *et al*. 2003; Cordeiro *et al*. 2004; Patel *et al*. 2009; Glukhov *et al*. 2010; Lou *et al*. 2011). However, experimental data on transmural and regional electrophysiological characteristics in the murine heart are still limited. In this study we have combined transverse murine cardiac slices with high resolution optical mapping for systematic transmural and regional profiling of both *V*_m_ and CaT across the murine ventricles. Our results indicate the presence of transmural APD heterogeneities within the wall of the left ventricle (Fig. 3), with the shortest APD observed in the epicardium, in agreement with previously published results for single isolated myocytes (Brunet *et al*. 2004) and ventricular tissue (Knollmann *et al*. 2001). However, no significant apico‐basal APD gradients were found in our study (Fig. 3), in contrast with previously reported findings on single isolated myocytes (Xu *et al*. 1999; Bondarenko *et al*. 2004). Transmural and apico‐basal gradients in the transient outward K^+^ current (*I*_to_) are thought to be the main determinant of APD heterogeneities in the mouse ventricle (Xu *et al*. 1999; Brunet *et al*. 2004; Rossow *et al*. 2006).

Our results also indicate the presence of significant interventricular differences in APD, with shorter APD in the right ventricle in comparison to the left ventricle and ventricular septum (Fig. 3). This is in agreement with data from rodent studies (Watanabe *et al*. 1983; Knollmann *et al*. 2001) and larger mammals investigations (Di Diego *et al*. 1996; Volders *et al*. 1999), and it is likely to be the result of differences in repolarisation currents which are significantly higher in cells isolated from the right than the left ventricle (Brunet *et al*. 2004; Molina *et al*. 2016).

Analysis of Ca^2+^ transients revealed the presence of transmural and regional heterogeneity of calcium handling in the murine ventricles, in addition to action potential gradients. A significant transmural gradient of CaTD50 and CaTD75 was found in the LV wall, with a longer CaTD in the endocardium compared to the epicardium (Fig. 4). Similar results have been reported for hearts of other species (Figueredo *et al*. 1993; Laurita *et al*. 2003). Regional differences in the expression of calcium regulatory proteins could explain the observed transmural CaTD heterogeneity. Differences in sarcoplasmic reticulum Ca^2+^‐ATPase (SERCA2a) and ryanodine receptor type 2 (RyR2) protein expression, and sodium–calcium exchanger function, have been reported between epicardial and endocardial cells in different species including mouse (Laurita *et al*. 2003; Cordeiro *et al*. 2004; Dilly *et al*. 2006; Lou *et al*. 2011). We also observed interventricular differences in CaTD50 in our study, with a shorter CaTD50 in the right ventricle in comparison to the left ventricle and ventricular septum (Fig. 4), consistent with previously reported interventricular differences in Ca^2+^ handling and contractility in rodent hearts (Kondo *et al*. 2006; Molina *et al*. 2016).

### AP and CaT alternans during rapid pacing

Cardiac alternans in the form of T‐wave alternans in the ECG, corresponding to beat‐to‐beat alternations in ventricular repolarisation, has demonstrated clinical utility in stratifying risk for malignant arrhythmias and sudden cardiac death, providing guidance for antiarrhythmic therapy (Verrier *et al*. 2011). At the cellular level, cardiac alternans manifests as rate‐dependent beat‐to‐beat alternations in contraction, AP morphology and CaT amplitude (Kanaporis & Blatter, 2015). In our study, we observed alternans in AP amplitude as well as in CaT amplitude when murine slices were electrically stimulated at high pacing rates (Figs 5 and 6). Alternans in AP amplitude have been reported previously in left ventricular wedge preparations from normal and post‐infarction rabbit hearts during rapid pacing (Myles *et al*. 2011). Importantly, alternans in AP amplitude were related to the development of arrhythmias during rapid pacing in the rabbit wedge, indicating that amplitude alternans may be an important mechanism for ventricular arrhythmia (Myles *et al*. 2011). As we demonstrated in Figs 5, 6, 7, arrhythmia was clearly triggered by high pacing frequency, when we observed reduced conduction velocities and areas of steep activation gradients. The majority of the events we observed were the non‐sustained ventricular tachycardia (VT) form; such non‐sustained VT is likely to be caused by focal activity and steep activation gradients.

Our results indicate the presence of significant transmural differences in pacing‐induced AP and CaT alternans in the mouse LV free wall, with greater incidence of alternans in the endocardium compared with the epicardium at both 8 Hz and 16 Hz pacing frequencies (Figs 5 *B* and 7 *B*). To the best of our knowledge, this is the first report describing transmural heterogeneities in frequency‐dependent AP and CaT alternans in the murine heart. Regional differences in CaT alternans have been previously described in the canine LV during rapid pacing in studies using wedge preparations and isolated myocytes (Laurita *et al*. 2003; Cordeiro *et al*. 2007). In these published studies, larger levels of alternans were observed in cells near the endocardium compared with cells near the epicardium (Laurita *et al*. 2003; Cordeiro *et al*. 2007), consistent with the findings of our study.

In the current study, we observed similar transmural and regional distributions of AP and CaT alternans. Electrical and CaT alternans are known to be highly correlated, although it remains controversial whether the primary cause of cardiac alternans is a disturbance of intracellular Ca^2+^ signalling or electrical membrane properties. In a recent study by Kanaporis and Blatter (2015), the mechanisms of calcium cycling and action potential dynamics in cardiac alternans have been investigated in single rabbit atrial and ventricular myocytes using combined [Ca^2+^]_i_ and electrophysiological measurements. The findings indicate that suppression of Ca^2+^ release from the sarcoplasmic reticulum abolished APD alternans, supporting a central role for intracellular Ca^2+^ cycling in the development of cardiac alternans (Kanaporis & Blatter, 2015). Cardiac slices, simultaneously loaded with voltage‐ and Ca^2+^‐sensitive dyes, will allow us to examine the cellular mechanisms of cardiac alternans in a more representative multicellular model system.

### Study limitations

A limitation to the study is the possibility of injury and uncoupling; partial uncoupling (reduction in conductance) could increase the differences in APDs between epicardial and endocardial regions.

### Conclusions

We have developed and validated a robust experimental methodology which combines transverse ultra‐thin cardiac slices and high resolution optical mapping to enable systematic analysis of transmural and regional gradients in *V*_m_ and CaT across the entire murine ventricles.

## Additional information

### Competing interests

We confirm that none of the authors has any conflicts of interests on the submission form and in the manuscript.

### Author contributions

Q.W, K.G, R,C, G.N and S.P: acquisition, analysis and/or interpretation of data for the work; G.H and C.OS: analysis and/or interpretation of data for the work, M.L and P.C: conception and design of the work, manuscript writing and revising it critically for important intellectual content,; D.P, D.T, J,W and G.F: supporting the work and revising manuscript critically for important intellectual content. All authors have approved the final version of the manuscript and agree to be accountable for all aspects of the work. All persons designated as authors qualify for authorship, and all those who qualify for authorship are listed.
